# Health Impacts and Recovery From Prolonged Freshwater Exposure in a Common Bottlenose Dolphin (*Tursiops truncatus*)

**DOI:** 10.3389/fvets.2020.00235

**Published:** 2020-05-07

**Authors:** Alissa C. Deming, Noel L. Wingers, Debra P. Moore, David Rotstein, Randall S. Wells, Ruth Ewing, Matthew R. Hodanbosi, Ruth H. Carmichael

**Affiliations:** ^1^Marine Mammal Research Center, Dauphin Island Sea Lab, Alabama Marine Mammal Stranding Network, Dauphin Island, AL, United States; ^2^College of Veterinary Medicine, Mississippi State University, Mississippi State, MS, United States; ^3^Marine Mammal Pathology Services, Olney, MD, United States; ^4^Chicago Zoological Society's Sarasota Dolphin Research Program, Sarasota, FL, United States; ^5^Southeast Fisheries Science Center, National Marine Fisheries Service, U.S. Department of Commerce, Miami, FL, United States; ^6^Department of Marine Sciences, University of South Alabama, Mobile, AL, United States

**Keywords:** Gulf of Mexico, freshwater skin lesions, corneal edema, out-of-habitat, human interaction

## Abstract

Common bottlenose dolphins (*Tursiops truncatus)* exposed to freshwater or low salinity (<10 practical salinity units; PSU) for prolonged periods of time have been documented to develop skin lesions, corneal edema and electrolyte abnormalities, and in some instances they have died. Here we review a case of an out-of-habitat subadult, female common bottlenose dolphin that remained in a freshwater lake in Seminole, Alabama for at least 32 days. Due to concerns for the dolphin's health a rescue was initiated. At the time of rescue bloodwork results indicated minor electrolyte abnormalities (hyponatremia, hypochloremia, hypoosmolality). Renal function was not affected (normal creatinine and urea nitrogen) and all other bloodwork parameters (hemogram; serum biochemistry analytes) were within normal limits. The dolphin was deemed healthy enough for immediate relocation and release. A satellite-linked tag was attached to the dorsal fin to track the dolphin following its relocation to a nearby brackish water bay (Perdido Bay, AL), a known habitat for bottlenose dolphins. Twelve weeks following release, the dolphin was found dead as a result of a fisheries interaction (peracute underwater entrapment). A full necropsy was conducted and there was complete resolution of the skin pallor and skin lesions and no evidence of chronic renal or central nervous system lesions. Post-mortem analysis of vitreous humor (used as a proxy for serum analytes and to determine post-mortem interval) was challenging to interpret and has not been validated in dolphins. This supports the need for future research in cetaceans to establish a species-specific approach. Elevated barium (Ba) concentrations in tooth dentin corresponded to increased seasonal freshwater discharge patterns, confirming repeated annual exposure to low salinity conditions prior to death and indicating freshwater exposure may pose an ongoing threat to dolphins in the region. This case provides a unique opportunity to follow the progression of prolonged freshwater exposure and recovery in a bottlenose dolphin and highlights that dolphins in nearshore habitats face a combination of persistent natural and human associated threats.

## Introduction

Prolonged exposure to freshwater or low salinity water (<10 practical salinity units; PSU) in common bottlenose dolphins (*Tursiops truncatus*; hereafter referred to as dolphins) has been recognized to cause negative health impacts, and in some cases, death (1–4). Dolphins have evolved to live in a marine environment where salinities maintain above 30 PSU. Cetaceans have developed the ability to excrete strongly hypertonic urine to conserve water and aid in fluid homeostasis in a marine environment (5–7). Some populations of dolphins that reside in bays, sounds and estuaries are known to be exposed to lower salinity water (15–25 PSU) and, on occasion, experience short periods of lower salinity (<15 PSU) as a result of freshwater runoff from watersheds, rivers and large storm events [e.g., floods and hurricanes; (3, 8, 9)]. Recent studies in Barataria Bay, Louisiana found that dolphins exhibited a preferred salinity threshold of >8 PSU and avoided waters of <5 PSU (10). Little is known about the effects of prolonged freshwater exposure in dolphins, including the length of exposure time necessary to cause negative health impacts and the ability of a dolphin to recover following prolonged low salinity exposure.

Exposure to excessive freshwater causes a range of pathologies in mammals. In terrestrial mammals, freshwater intoxicosis can occur if large quantities of freshwater are consumed in a short period of time as seen in psychogenic polydipsia (described in canines, hoofstock, and humans), self-imposed overhydration in Army recruits following heat-related injuries, iatrogenic causes (as seen in fluid overloading with intravenous fluids in hospitalized animal and human patients) and child abuse (11–16). In humans, the early stages of water intoxication include confusion, disorientation and vomiting, with progressive exposure resulting in hyponatremia, seizures, coma, and death (15). In wild dolphins, prolonged exposure to freshwater causes generalized skin pallor, various degrees of proliferative, erosive and ulcerative skin lesions with possible secondary bacterial, fungal and protozoal infections, corneal edema, and electrolyte imbalances (including hyponatremia, hypochloremia, and low calculated osmolality) (1, 3, 4). In cases of freshwater exposure and dolphins, however, there is little documentation of the clinical signs antemortem or the pathophysiology post-mortem, primarily due to the lack of *a priori* knowledge of freshwater exposure of stranded dolphins and the availability of fresh post-mortem specimens to examine histologically.

Several approaches to determine whether a dolphin had been exposed to prolonged freshwater prior to death have been proposed or are currently being investigated. These approaches include the use of vitreous humor post-mortem as a proxy for serum electrolytes and trace elements analyses of teeth, which can have distinct freshwater and marine patterns (4, 6, 17–19). Currently the most common means to assess freshwater exposure in stranded dolphin carcasses is evaluation of the skin for lesions consistent with prolonged freshwater exposure and known exposure to low salinity water (1, 3).

To date, the vast majority of prolonged freshwater exposure reports in dolphins have been associated with out-of-habitat dolphins that traveled to and remained in freshwater systems for prolonged periods of time or were displaced as a result of natural disasters such as flooding or hurricanes (1, 3, 9). In the Southeast United States from 1992 to 2005, there were 12 documented cases of bottlenose dolphins that were classified as out-of-habitat because they were free-swimming in low salinity or freshwater systems for prolonged periods of time (1). Six of the 12 cases resulted in death attributed to prolonged freshwater exposure and the remaining cases required lifesaving intervention (rescue, relocation, or rehabilitation). Due to a limited understanding of how long dolphins can tolerate exposure to low salinity water, it can be challenging for authorities to determine at what point life-saving intervention is necessary for out-of-habitat dolphins. Stranding responders and authorities often encourage dolphins to leave an area through various herding techniques and monitor for signs of decreased foraging, skin pallor, or skin lesions. Once a dolphin is deemed to be in distress, rescue, and relocation or rehabilitation is pursued. Most of the knowledge of the physiological impacts of prolonged freshwater exposure has been gained during these efforts (1, 4). Following rescue and relocation, stranding responders attempt to track the dolphin to determine success of the rescue, but there is limited information in the literature about the ability of a dolphin to recover from prolonged low salinity exposure. Here we review a case of a common bottlenose dolphin that recovered following rescue from prolonged freshwater exposure but ultimately died from human interaction. This case gives a unique opportunity to assess the progression and resolution of freshwater skin lesions following relocation to more appropriate habitat conditions.

## Case Description and Analytical Methods

**Figure 1:**
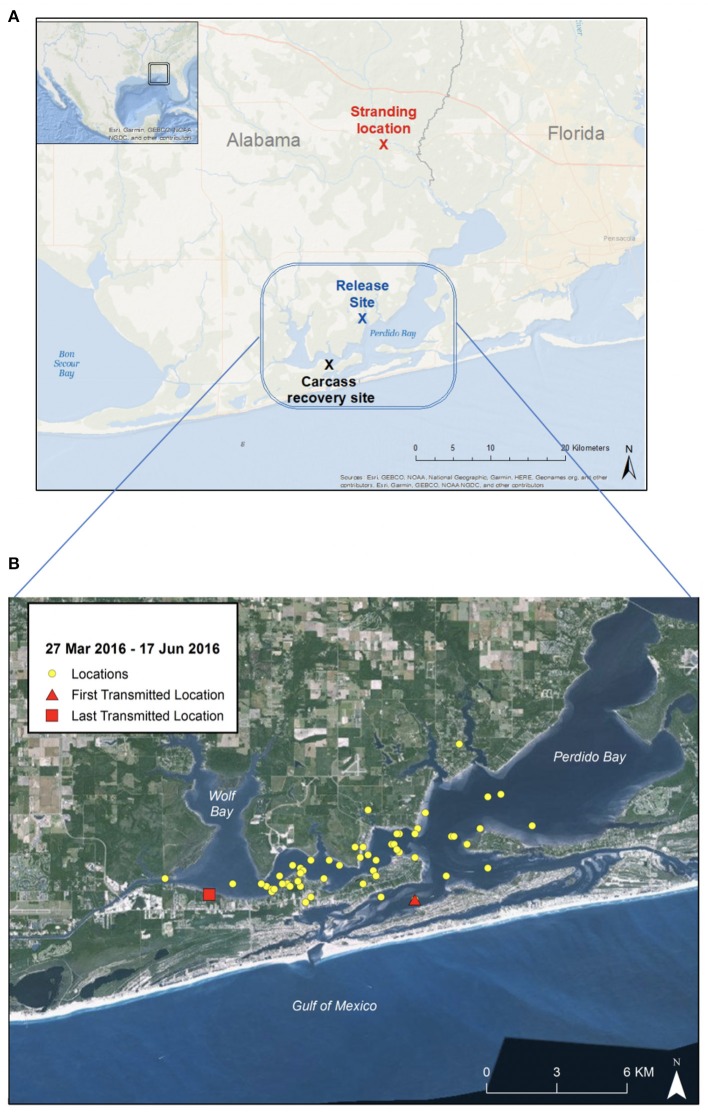
Maps of Perdido Bay, Alabama. **(A)** Rescue location (red), release site (blue), and site of carcass recovery (black) with blue rectangle region indicating area dolphin spent time during 12-week recovery period. **(B)** Close-up map of Perdido Bay showing satellite tracking signal locations (27 March 2016 to 17 June 2016). The dolphin remained within Perdido Bay, a known estuarine habitat for bottlenose dolphins, and did not appear to travel south into the Gulf of Mexico during the post-release period.

**Figure 2:**
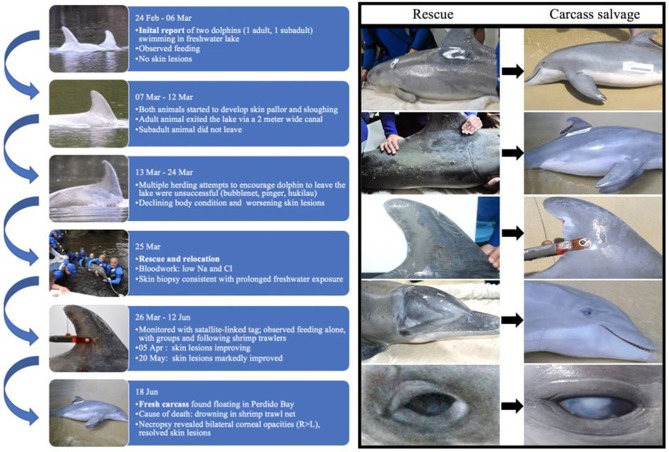
Timeline of case from initial report (24 February 2016) to time of death (18 June 2016) and photographs of skin lesions taken at time of rescue (25 March 2016; left column **A–E**) and time of carcass recovery (18 June 2016; right column **F–J**). During the 12-week period following rescue from a freshwater lake and release into a brackish water estuary, skin lesions markedly improved, with almost complete resolution. Corneal opacities were worse at time of death than at time of rescue. Left lateral body **(A,F)**; right flank **(B,G)**; right dorsal fin **(C,H)**; right lateral head **(D,I)**; and right eye **(E,J)**.

On 23 February 2016, a member of the public reported to the Alabama Marine Mammal Stranding Network (ALMMSN) that two dolphins (suspected mother with older calf) were swimming in a small freshwater lake in Seminole, Alabama (30.502990°N, −87.442926°W) (Figure 1A-map; Figure 2-timeline of case). The complainant provided photos of the dolphins and reported they were traveling together in the lake, observed foraging and appeared healthy. The lake is connected to the Perdido Bay estuary system approximately 25-km south, via a small, shallow canal (Figure 1A). Perdido Bay (salinity average 15–25 PSU) is a region considered by the National Oceanographic and Atmospheric Association, National Marine Fisheries Service (NOAA/NMFS) to be suitable habitat for bottlenose dolphins, and NOAA/NMFS is responsible for their management (20). Under the direction of NOAA/NMFS, the dolphins were monitored with anticipation that they would leave the area on their own volition.

Thirteen days later (07 March 2016), the dolphins were still in the lake and were no longer observed feeding. On 08 March 2016, ALMMSN staff assessed the dolphins onsite and both had mild erosive skin lesions and slight generalized skin pallor. Otherwise they appeared to be in good body condition and behaving normally. During monitoring, the larger dolphin exited the lake through a 2-meter wide canal that connected to Perdido River and southward to Perdido Bay. The larger dolphin was not observed in the lake again and the fate of this dolphin is unknown. The smaller dolphin remained in the lake. On 13 March 2016 ALMMSN, Emerald Coast Wildlife Refuge (ECWR), and NOAA/NMFS responders attempted to herd the remaining dolphin from the lake using bubblenet, pingers, and a hukilau, but efforts were unsuccessful. Due to the decrease in observed foraging, deteriorating body condition and worsening skin lesions, officials deemed the dolphin was in need of rescue and either relocation or rehabilitation if indicated at time of rescue.

On 25 March 2016, ALMMSN, regional collaborators, and NOAA/NMFS initiated a rescue (Figure 2). The dolphin was captured using a boat-based, net capture technique (21–23). Once secured, the dolphin was brought to land and veterinarians performed a thorough physical examination, collected blood samples (heparinized whole blood and serum) from the ventral fluke vessel, skin culture (fungal only), and skin biopsies for histologic evaluation and molecular analysis. Fungal skin culture swabs were collected from an area with moderate skin sloughing (left and right dorsolateral thorax). Local anesthesia (lidocaine with epinephrine; L-block) was administered and two skin/blubber biopsies extending the full thickness of the blubber were collected with an 8-mm biopsy punch, near the location of culture swab collection. The skin/blubber biopsies were cut in half longitudinally and one section of each was fixed in 10% neutral buffered formalin for histologic evaluation and the remaining sample was frozen for fungal culture and/or molecular analysis as needed (−20°C). Following skin biopsy fixation, the tissues were embedded in paraffin, sectioned at 5-μm thickness, stained histochemically with hematoxylin and eosin (H&E), gram, giemsa, acid fast bacteria (AFB) stain, Grocott's methenamine silver (GMS) stains, and evaluated by a veterinary pathologist.

Point of care electrolyte analysis (iStat CG8+) was performed on heparinized whole blood (Table 1), which showed a mild hyponatremia (146 mEq/L; reference range: 149–157 mEq/L) and hypochloremia (98 mEq/L; reference range: 110–119 mEq/L) with normal creatinine (0.9 mg/dL; reference range: 0.68–1.49 mg/dL) and low urea nitrogen (34 mg/dL; reference range: 42–77 mg/dL) (24). Hemogram and serum biochemistry profiles were also performed, and with the exception of the above-mentioned electrolyte abnormalities, all other parameters were within normal limits for a wild subadult bottlenose dolphin (24). It was determined that the dolphin was in suitable condition for immediate relocation and release. The dolphin was transported by vehicle to the mouth of Palmetto Creek (30.34691°N, −87.51409°W) in Perdido Bay, AL (Figure 1A). Salinity at the release location was 6.2 PSU. A satellite-linked SPOT-299B Single-point Finmount, 2-Lay tag (Wildlife Computers, Redmond, WA, USA) was applied to the trailing edge of the dorsal fin and the dolphin was released (23, 26). The tag was programmed to transmit 250 times each day, during five periods of suitable satellite overflight. Post-release, the dolphin's movements were monitored via satellite-linked tag transmissions, boat observations and communication with local dolphin watching tour operators. Throughout the 85-day tracking period the dolphin remained in Perdido Bay (Figure 1B). During this time, salinity levels measured at the closest environmental monitoring buoy in Bon Secour Bay (Alabama Real-time Coastal Observing System; https://arcos.disl.org/stations/disl_stations?stationnew=106) were relatively low, particularly at the time of relocation (3.7 PSU on 25 March 2016), and steadily increased throughout the post-release period (18.5 PSU on 18 June 2016; **Figure 4**).

**Table 1:** Biochemical analytes of serum (red) from apparently healthy wild Atlantic juvenile bottlenose dolphins (24); and biochemical analytes from this case at time of rescue (serum) and post-mortem (vitreous).

**Biochemical analyte**	**Healthy (24)**	**Rescue (this case)**	**Post-mortem (this case)**
Sodium (mEq/L)	149–157	147	145 (149.4)*
Chloride (mEq/L)	110–119	96	123 (127.4)*
Potassium (mEq/L)	3.2–4.4	4.2	41.5
Creatinine (mg/dL)	0.68–1.49	0.9	1.7
Urea Nitrogen (mg/dL)	42–77	34	104

* *Value was corrected using potassium to determine a post-mortem interval of 48 h (25) and applying the post-mortem sodium and chloride correction factors established by Zilg et al. (19)*.

On 18 June 2016, ALMMSN received a call from Orange Beach Coastal Resources regarding a fresh-dead dolphin with a satellite-linked tag on the dorsal fin, floating in the surf (Orange Beach, AL; 30.301585°N, −87.559443°W; Figure 1A). ALMMSN responded, took photographs (Figure 2), and transported the carcass to Dauphin Island Sea Lab. The carcass was placed in a refrigerated cold room (4°C) overnight, and on 19 June 2016 a necropsy was performed. The carcass was fresh dead (Code 2) at time of salvage and in early decomposition (Code 3, early) at the time of necropsy. A full suite of tissues was collected and fixed in 10% neutral buffered formalin. Following fixation, the tissues were embedded in paraffin, sectioned at 5-μm thickness, stained with H&E, and evaluated by a pathologist. Additionally, a small piece of green net material caught in the right attachment screw of the satellite-linked tag and a small hook found free-floating in the forestomach were submitted to the NOAA/NMFS Gear Research Team for analysis.

At necropsy, 24-h following the carcass report, vitreous humor was collected to serve as a proxy for serum (19, 27, 28). The vitreous was red-tinged, indicating possible blood contamination, most likely related to retinal or fundic hemorrhage associated with drowning/asphyxiation (29). Vitreous was centrifuged for 10 min at 1,800 g and the liquid supernatant collected, frozen (−20°C) and shipped overnight on dry ice to Cornell University's Animal Health Diagnostic Center for electrolyte analysis. Results were compared to published references for serum chemistry analytes in wild juvenile bottlenose dolphins (24). There was no urine in the bladder for urinalysis.

Because this dolphin experienced a known period of prolonged freshwater exposure, trace element analysis and age estimation were performed on a tooth to determine if a freshwater elemental signature could be identified in the most recent growth layer group (GLG) (17, 18, 30–32). For trace element analysis, a 2-mm mid-sagittal tooth section was cut using a Buehler IsoMet 1000 Precision saw with a diamond blade. Trace element data were collected along the cut surface through laser ablation inductively coupled plasma mass spectrometry (LA-ICPMS) using a NWR213 Nd:YAG laser coupled with an Agilent 7700x quadrupole ICPMS. Trace element values for ^43^Ca, ^137^Ba, ^208^Pb were collected. Laser scan parameters were set as 30 μm s^−1^ scan speed, 10 μm spot size at 20 Hz, with ablation fluence of 45% (15–17 J cm^−2^). SRM612 glass (National Institute of Standards and Technology) and MAPS-4 phosphate pellet (United States Geological Survey) were used as tuning and reference standards, respectively. The concentrations of all elements were normalized to ^43^Ca, and data were analyzed using 2-point reference calibration in Microsoft Excel. To relate trace element ratios to the time of elemental incorporation, we estimated the dolphin's age using established sclerochronological age determination methods (31, 32). Briefly, the 2-mm tooth section was decalcified with RDO decalcifying solution, then further sectioned into 25-μm slices. These slices were stained with a hematoxylin solution and placed onto microscope slides. Age was estimated by counting the number of GLG in the post-natal dentin of the tooth (31, 32).

## Diagnostic Assessment

**Figure 3:**
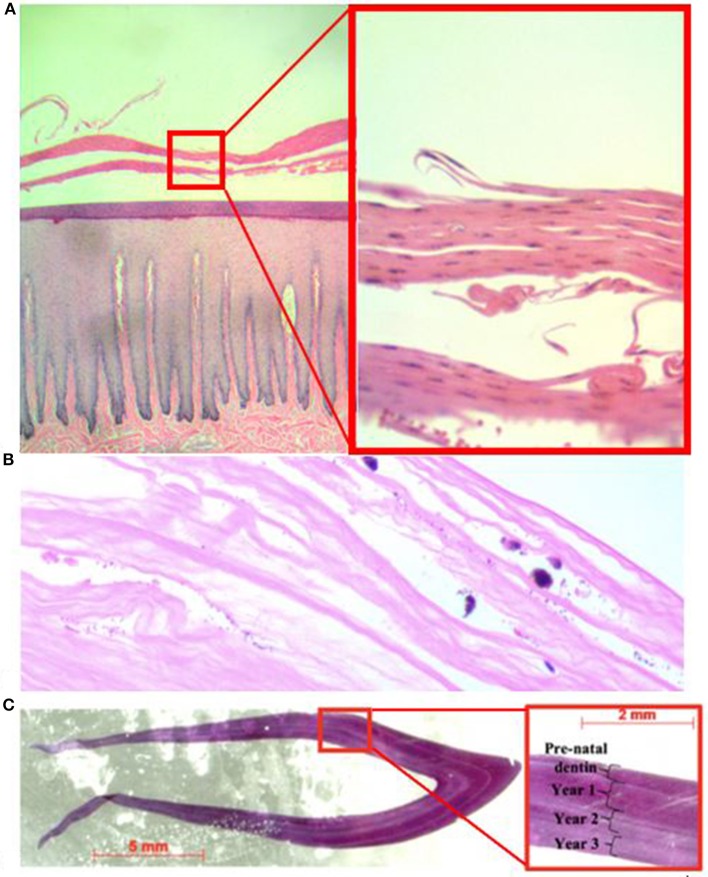
Photomicrograph of hematoxylin and eosin stained tissue slides including **(A)** skin biopsy at time of rescue (left = 4X; right = 20X). The stratum externum is separated by eosinophilic fluid and epithelium is separated and curled; **(B)** corneal edema of the right eye at time of necropsy (20X); and **(C)** cross-section of tooth used for trace element analysis and close-up (red box) showing annual growth layer groups used to estimate the dolphin's age.

At the time of rescue, following 32 days of freshwater exposure, the dolphin was slightly thin. The skin was diffusely pale grey and sloughing (>70%) with superficial erosive lesions on the flukes, head, mid-epaxial region, and dorsal fin (Figure 2). Skin biopsies collected at time of rescue were examined histologically and there was epithelial erosion, edema, hydropic degeneration of superficial epithelial cells, and separation of superficial epithelial layers with serum accumulation (Figure 3A). Special stains (H&E, gram, giemsa, AFB, GMS) for fungi, bacteria and protozoa were negative, and no fungal hyphae were identified. Fungal culture grew *Curvularia* sp. (Fungus Testing Laboratory, UT, USA), a pigmented environmental hyphomycete found in the soil and plant debris, but fungal PCR from frozen skin biopsy was negative. The organism was likely a superficial saprophyte and was not present within the epidermal layers.

When the carcass was recovered, 85 days after being relocated to brackish water (range = 3.7–20.7 PSU; mean = 13.4; *SD* = 4.4 PSU), the freshwater skin lesions were markedly improved to completely resolved (Figure 2) and the epidermis was normal on histologic evaluation. The most significant finding on necropsy included a small piece of net remnant on the satellite-linked tag, several minimally digested fish and invertebrates in the forestomach, and superficial criss-cross linear impressions on the skin of the rostrum, melon and leading edges of the pectoral fins and flukes. Additional gross findings included a moderate amount of red-tinged froth in the bronchi and bronchioles, occasional petechiae, and ecchymosis on the intestinal mucosa, a free-floating bait hook in the forestomach and a mild to moderate trematode (*Braunina cordiformis*) burden in the main (glandular) stomach. Both eyes had mild (left) to marked (right) corneal opacities (Figure 2), that were worse at time of carcass recovery compared to time of rescue and had moderate corneal edema on histologic evaluation (Figure 3B).

The majority of histologic findings were considered incidental and included few protozoal cysts (*Sarcocystis* sp.) in the skeletal muscle without an inflammatory response, mild lymphoplasmacytic pneumonia and pulmonary fibrosis and gastric *B. cordiformis*. There were no histopathologic lesions in the kidneys, integument, adrenal glands or central nervous system that would indicate prolonged or chronic effects associated with the freshwater exposure. The net material evaluated by NMFS Gear Research Team was identified to be trawl net #7 or #9 (twisted nylon twine) that had been dipped and is used in inshore recreational and commercial shrimp trawls. The small hook recovered from the forestomach was a 3/0 offset live bait hook, sometimes called a “Mosquito Hook.” Cause of death was peracute underwater entrapment in a shrimp trawl net supported by the presence of net material and criss-cross net impression on the body along with tracheobronchial froth and enteric petechiae (33).

The post-mortem electrolyte analysis of the vitreous had a severely elevated potassium (41.5 mEq/L), mildly low sodium (145 mEq/L), mildly low chloride (123 mEq/L), mildly elevated creatinine (1.7 mg/dL), and mildly elevated urea nitrogen (104 mg/dL). Based on equations developed in human forensics using vitreous potassium levels (34), the post-mortem interval (PMI) was estimated to range from 42 h [(PMI = 1.076(K^+^)−2.815 with K = 41.5 mEq/L; (25)] to >120 h [(PMI = 6.15(K^+^)−38.1 with K = 41.5 mEq/L; (35)] at time of vitreous collection. Post-mortem changes in sodium and chloride of 2.2 mEq/L every 24 h following death have been documented in humans (19) and represent the best available data for making similar estimates in marine mammals. To apply Zilg et al. (36) sodium and chloride correction factor, we used the conservative PMI estimate of 42 h and rounded up to 48 h to enable application of the sodium and chloride correction factor, which is calculated for 24-h intervals. This estimates the vitreous sodium at time of death to be 149.4 mEq/L and chloride equivalent to 127.4 mEq/L. No similar approaches have been developed to apply to PMI increases in creatinine or urea nitrogen.

**Figure 4:**
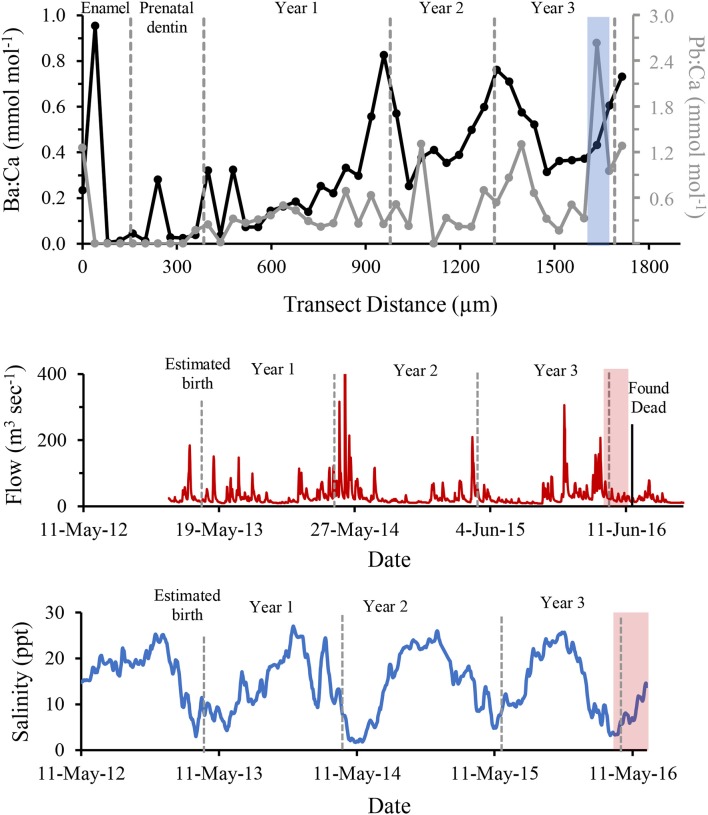
Ba (black) and Pb (grey) concentrations normalized to Ca and compared to years of life of a known freshwater-exposed dolphin, determined by LA-ICPMS across growth layer groups in a tooth **(Top)**. Freshwater discharge to the area from the Perdido and Styx Rivers is shown for the same time period **(Middle)**. Historical salinity levels measured at closest environmental monitoring buoy (Bon Secour) to relocation site **(Bottom)**. Dashed grey bars indicate boundaries of annual growth layer groups (shown in this figure), the period of known stranding within a freshwater canal is indicated by light blue shaded regions, and red shaded region marks period from relocation to time if death. Discharge data were obtained from publicly available United States Geological Survey data at monitoring sites USGS02377750 (Styx River, Seminole, AL) and USGS02376500 (Perdido River, Barrineau Park, FL). Salinity data were obtained from Alabama Real-time Coastal Observing System, Bon Secour monitoring station (https://arcos.disl.org/stations/disl_stations?stationnew=106).

Based on the number of GLGs present in the post-natal dentin, we estimate the dolphin was slightly over 3 years old at death (Figure 3C). Ontogenetic shifts occurred in Ba:Ca and Pb:Ca indicated by differences in elemental composition between enamel, pre-natal dentin and post-natal dentin (Figure 4, top). Post-natal peaks in Ba:Ca correlated with seasonal freshwater inputs to the system and salinity (Figure 4, middle and bottom, respectively). Peaks in Pb:Ca followed Ba:Ca, particularly in years 2–3, with a spike in Pb:Ca during the period of known freshwater exposure prior to death.

## Discussion

This report describes a case of prolonged freshwater exposure in a common bottlenose dolphin that remained in a freshwater lake for at least 32 days, prior to successful rescue and recovery. The prolonged freshwater exposure resulted in diffuse skin lesions, mild electrolyte abnormalities, corneal edema, and decreased body condition. Following 85 days in a brackish water bay with somewhat more appropriate salinity (range = 3.7–20.8 PSU; mean = 13.7; *SD* = 4.4), the dolphin was found dead as a result of a fisheries interaction (peracute underwater entrapment). At the time of death, freshwater-related skin lesions were resolved and there were no chronic renal or central nervous system lesions on histologic examination, but corneal edema worsened, likely affecting vision in at least one eye. It is difficult to determine if the corneal edema was sequelae of the freshwater exposure, the result of trauma to the cornea either during the rescue capture, transport or during the 12-weeks following relocation, or some combination of these.

A typical treatment for corneal edema in dolphins managed under human care is topical application of hypertonic saline drops (5% NaCl). Therefore, moving a dolphin from freshwater to higher salinity brackish water should improve conditions relative to the corneal edema. However, it may be that the brackish water in Perdido Bay was still too hypotonic to allow the already compromised corneal epithelium to heal, and the corneal edema continued to worsen. This observation warrants close monitoring of dolphin eyes following transition between low and higher salinity water and stresses the importance of protecting the eyes during capture and transport. If a dolphin with prolonged freshwater exposure is brought into rehabilitation, application of hypertonic saline eye drops may be indicated to decrease corneal edema and likelihood of corneal fibrosis that could impact long-term vision. This case provides valuable insight into the health effects of a dolphin during a known period of freshwater exposure and supports that relocation to higher salinity habitat may enable successful recovery from some symptoms (skin lesions) but not others (corneal edema).

The analysis of tooth GLGs indicated this dolphin was 3–4 years old, which corresponds to the age class associated with its straight length [190 cm; (37)]. The observed seasonal increases in Ba:Ca in the tooth coincided with seasonal pulses of low-salinity water and is consistent with expected elevated Ba content in freshwater. A negative correlation between Ba:Ca in water and salinity has been shown for the northern Gulf of Mexico (38), and thus, Ba:Ca ratio is often used as an indicator of freshwater residency for a variety of finfish (39–41). Our data demonstrate that this application may be effective in dolphins to trace regular seasonal exposure to freshwater. The elevated Pb:Ca values at the time of acute freshwater exposure in the enclosed lake indicate the dolphin was exposed to higher concentrations of Pb, either conveyed in freshwater discharge or through consumption of contaminated fish (42). This finding suggests freshwater has potential to affect dolphin health through increased exposure to local anthropogenic contaminants, as seen elsewhere in the southeastern U.S. (43), and merits further study. Importantly, these data suggest resident dolphins along the northern Gulf of Mexico coast repeatedly endure at least seasonal low-salinity conditions and exposure to elements conveyed via freshwater discharge throughout their lifetime.

The electrolyte analysis on the post-mortem vitreous humor is challenging to interpret. There is a lack of validation using this technique in cetaceans, and there can be significant variability associated with ante- and post-mortem factors (including autolysis, ambient temperature, drowning in low salinity water, blood contamination, and/or corneal disease potentially impacting the permeability of the cornea- all potential confounding variables in this case). Vitreous analytes in humans and some animals (cows, dogs, and cats) have been used to determine the PMI, specifically using potassium. Vitreous humor acts as a proxy for antemortem serum chemistry analyte analysis including sodium, chloride and nitrogenous compounds (creatinine and urea nitrogen) (19, 27, 44, 45). It is generally accepted that potassium, urea nitrogen, and creatinine increase with increasing PMI (27, 34, 44, 45), and sodium and chloride decrease with increasing PMI (19). Using the calculated PMI, the vitreous sodium was corrected using the method described by Zilg et al. (19), which gave a sodium value of 149.4 mEq/L (within normal limits). However, chloride appeared elevated both before and after applying the correction factor. The slight elevation in vitreous creatinine and urea nitrogen may reflect a post-mortem increase as seen in other species, or could be the result of antemortem abnormalities, possibly associated with chronic renal disease and/or rhabdomyolysis associated with struggle prior to death. Due to the various confounding variables with this case and lack of established methodology in cetacean species, the error associated with these values cannot be quantified at this time. Future work on post-mortem vitreous analytes in cetacean species is needed and has the potential to serve as a useful tool in confirming prolonged freshwater exposure once established.

Post-mortem evaluation determined the cause of death to be drowning in a shrimp trawl net. Although the freshwater effects on the skin improved through time, visual impairment secondary to prolonged freshwater exposure may have resulted from progressive corneal edema, perhaps connected to interactions with fisheries for easier prey procurement, resulting in death. The combination of natural and anthropogenic influences on the life of this animal alludes to a larger story. Combined threats from anthropogenically-exacerbated influences, such as climate-related changes to rainfall or pulse storm events, and increased fishery pressures or freshwater diversion, have the potential to synergistically impact vulnerable bay, sound, and estuary bottlenose dolphin populations. These factors may further cause animals to stray out-of-habitat or endure changes to habitat that make it less suitable. This study highlights the likelihood of these multiple or cumulative stressors to negatively affect nearshore dolphins.

## Data Availability Statement

The datasets generated for this study are available on request to the corresponding author.

## Ethics Statement

This animal study was reviewed and approved by National Oceanographic and Atmospheric Association, National Marine Fisheries Service under a Stranding Agreement issued to the Dauphin Island Sea Lab.

## Author Contributions

All contributing authors have approved this work for publication. Individual contributions were distributed as follows: AD, NW, DM, DR, RE, RW, MH, and RC: study concept and design, sample and data acquisition, and data interpretation. AD, DR, RW, RE, and RC: manuscript preparation. NW, DM, and MH: manuscript review.

## Conflict of Interest

The authors declare that the research was conducted in the absence of any commercial or financial relationships that could be construed as a potential conflict of interest.
